# Supporting dataset on the optimization and validation of a QuEChERS-based method for the determination of 218 pesticide residues in clay loam soil

**DOI:** 10.1016/j.dib.2020.106393

**Published:** 2020-10-09

**Authors:** Andrea Acosta-Dacal, Cristian Rial-Berriel, Ricardo Díaz-Díaz, Mª del Mar Bernal Suárez, Manuel Zumbado, Luis Alberto Henríquez-Hernández, Octavio P. Luzardo

**Affiliations:** a Toxicology Unit, Research Institute of Biomedical and Health Sciences (IUIBS), Universidad de Las Palmas de Gran Canaria, Paseo Blas Cabrera s/n, 35016 Las Palmas de Gran Canaria, Spain; b Department of Environmental Analysis, Technological Institute of the Canary Islands, C/ Los Cactus no 68 35118, Polígono Industrial de Arinaga, Agüimes, Las Palmas, Canary Islands, Spain; c Spanish Biomedical Research Centre in Physiopathology of Obesity and Nutrition (CIBERObn), Paseo Blas Cabrera Felipe s/n, 35016 Las Palmas, Spain

**Keywords:** Pesticides, Soils, QuEChERS, LC-MS/MS, GC–MS/MS, Method optimisation, Matrix effect

## Abstract

The dataset presented in this article supports “Optimization and validation of a method for the simultaneous environmental monitoring of 218 pesticide residues in clay loam soil” [1]. A method based on QuEChERS (Quick, Easy, Cheap, Effective, Rugged & Safe) for the extraction of pesticide and some metabolites residues was developed. The quantification of the chemicals was performed by a combination of two complementary LC-MS/MS and GC–MS/MS analyses. Detailed optimization data of the QuEChERS extraction method is provided, including (1) salt combination, (2) acidification of the solvent (3) the amount of the selected acid (Formic Acid, FA) and (4) moisturization of the soil samples prior to extraction. In addition, all the validation data are presented, including the matrix effect, which was evaluated for each analyte using the recommended procedure.

## Specifications Table

**Table:** 

Subject	Environmental Chemistry
Specific subject area	Pesticide extraction in agricultural soils for LC/MS-MS and GC–MS/MS analysis
Type of data	Tables and figures (processed data), and the corresponding raw data
How data were acquired	Ultra-high performance liquid chromatography coupled to triple quadrupole mass spectrometry (LC-MS/MS), models 1290 (UHPLC)-6460 (MS/MS). Agilent Technologies, Palo Alto, CA, USA Gas chromatography coupled to triple quadrupole mass spectrometry (GC–MS/MS), models 7890B (GC)-7010 (MS/MS). Agilent Technologies, Palo Alto, CA, USA.
Data format	Raw and analysed
Parameters for data collection	Firstly, the selection of the salts for the first step of the method based on QuEChERS (Quick, Easy, Cheap, Effective, Rugged & Safe) in development were assessed comparing the well-know AOAC and EN procedures. During this step, the possible inclusion of a clean-up step was studied with Enhance Matrix Removal (EMR, Agilent Technologies). Next, a comparison of the efficiency of acidification of the extraction solvent was performed. The acetonitrile was acidified with acetic acid (AA) or formic acid (FA) and compared to non-acidified acetonitrile. Then, the optimization of the percentage of the selected acid, FA, to be added to the extraction solvent was studied. Additionally, the potential improvement on the recoveries of the addition of water to the sample prior to extraction was also tested. Finally, the matrix effect of the analytes in LC-MS/MS and GC–MS/MS was assessed following the recommended procedure.
Description of data collection	The method selection and the optimization experiments were performed at a single concentration of 20 ng g^−1^ (in triplicate). Blank soil samples were spiked with the 218 pesticides in the different conditions tested: (1) AOAC (American Association of Official Analytical Chemists) vs. EN (European Norm) QuEChERS salts with and without clean-up step (2) extraction solvent acidification: acetonitrile vs acetonitrile-1% acetic acid vs 1% acetonitrile-formic acid (3) amount of formic acid in the solvent: 0.5%, 1% and 2.5% of formic acid and (4) moisture of the soil samples: dry samples, 10%, 20%, 30%, 40% and 50% of moisture. The matrix effect was evaluated by a comparison of the response of a mixture of the 218 pesticide standards in the soil matrix extracted with the developed method and the signal obtained for the standards in the solvent (2.5% FA-ACN) at the same concentration, 50 ng mL^−1^ (in triplicate). Both samples and standards in matrix were injected in the liquid chromatographer diluted with water (1:1) for all experiments. In the matrix effect experiments, solvent for LC-MS/MS was water-acetonitrile-2.5%F.A. The chromatographic and mass spectrometry data for both LC-MS/MS and GC–MS/MS was analysed with MassHunter Quantitative Analysis software (Agilent Technologies).
Data source location	Institution: Toxicology Unit, Clinical Sciences Department, Universidad de Las Palmas de Gran Canaria City/Town/Region: Las Palmas de Gran Canaria (Gran Canaria, Canary Islands) Country: Spain
Data accessibility	With the article
Related research article	Andrea Acosta-Dacal, Cristian Rial-Berriel, Ricardo Díaz, María del Mar Bernal Suárez. Optimization and validation of a method for the simultaneous environmental monitoring of 218 pesticide residues in clay loam soil. Science of the Total Environment, 2020, 753, 142,015. DOI: 10.1016/j.scitotenv.2020.142015

## Value of the Data

• The optimization data might be useful to other researchers developing QuEChERS-based extraction methods in soil matrix.
• The validated data provided are equally useful for researchers developing methods in other matrices of similar complexity.
• The details of the matrix effect of each analyte demonstrates the need of using matrix-matched calibration curves in order to counteract ion suppression or enhancement in chromatography-triple quad mass spectrometry tandems, especially in GC–MS/MS.

## 1 Data Description

The data presented here were obtained during the development and validation of a QuEChERS-based extraction method for the detection and quantification in GC–MS/MS and LC-MS/MS of 218 pesticides in soil matrices and supports the main article in Science of the Total Environment entitled “Optimization and validation of a method for the simultaneous environmental monitoring of 218 pesticide residues in clay loam soil” [1].

Table 1 is a list of the analytes presented in alphabetical order together with an identification number from 1 to 218 and the technique in which they are analysed. Thus, compounds are identified numerically with their correspondent label in the following charts.

**Table 1:** List of compounds analysed through the optimization process.

Compound	Technique^a^	No.	Compound	Technique^a^	No.	Compound	Technique^a^	No.	Compound	Technique^a^	No.
4,4′-Dichlorobenzophenone (metabolite of dicofol)	GC	1	Dimethenamide	LC	56	Imidacloprid	LC	111	Prochloraz	LC	166
4,4′-Dicofol	GC	2	Dimethoate	LC	57	Indoxacarb	LC	112	Procymidone	GC	167
Abamectine	LC	3	Dimethomorph (two isomers)	LC	58	Iprodione	GC	113	Profenofos	LC	168
Acephate	LC	4	Diniconazole-M	LC	59	Iprovalicarb	LC	114	Propargite	LC	169
Acetamiprid	LC	5	Dinocap	LC	60	Isocarbophos	GC	115	Propiconazole	LC	170
Acrinathrin	LC	6	Diphenylamine	LC	61	Isofenphos methyl	LC	116	Propoxur	LC	171
Aldicarb	LC	7	Endosulfan alfa	GC	62	Isoprothiolane	LC	117	Propyzamide (pronamide)	LC	172
Aldicarb sulfone	LC	8	Endosulfan beta	GC	63	Kresoxim methyl	LC	118	Proquinazid	LC	173
Atrazine	LC	9	EPN	LC	64	Linuron	LC	119	Prothioconazole-desthio	LC	174
Azinphos methyl	LC	10	Epoxiconazole	LC	65	Lufenuron	LC	120	Prothiophos	GC	175
Azoxystrobin	LC	11	Esfenvalerate	GC	66	Malaoxon	LC	121	Pyraclostrobin	LC	176
Benalaxyl	LC	12	Ethion (diethion)	LC	67	Malathion	LC	122	Pyrazophos	LC	177
Bendiocarb	LC	13	Ethofumesate	GC	68	Mandipropamid	LC	123	Pyridaben	LC	178
Bifenthrin	GC	14	Ethoprophos	LC	69	Mefenoxam (metalaxyl-M)	LC	124	Pyridaphenthion	LC	179
Bitertanol	LC	15	Etofenprox	LC	70	Mepanipyrim	LC	125	Pyrimethanil	GC	180
Boscalid (formely nicobifen)	GC	16	Etoxazole	LC	71	Metaflumizone	LC	126	Pyriproxifen	LC	181
Bromopropylate	GC	17	Famoxadone	LC	72	Metalaxyl	GC	127	Quinalphos	LC	182
Bromuconazole (two isomers)	LC	18	Fenamidone	LC	73	Metaldehyde	LC	128	Quinoxyfen	LC	183
Bupirimate	LC	19	Fenamiphos	LC	74	Metconazole	LC	129	Rotenone	LC	184
Buprofezin	LC	20	Fenamiphos sulfone	LC	75	Methamidophos	LC	130	Simazine	LC	185
Cadusafos (ebufos)	LC	21	Fenamiphos sulfoxide	LC	76	Methidathion	LC	131	Spirodiclofen	LC	186
Carbaryl	LC	22	Fenarimol	GC	77	Methiocarb	LC	132	Spiromesifen	LC	187
Carbofuran	LC	23	Fenazaquin	LC	78	Methiocarb sulfone	LC	133	Spirotetramat	LC	188
Carbofuran-3-hydroxy	LC	24	Fenbuconazole	LC	79	Methiocarb sulfoxide	LC	134	Spirotetramat-enol	LC	189
Chlorantraniliprole	LC	25	Fenbutatin oxide	LC	80	Methomyl	LC	135	Spiroxamine (two isomers)	GC	190
Chlorfenapyr	GC	26	Fenitrothion	GC	81	Methomyl oxime	LC	136	Tebuconazole	LC	191
Chlorfenvinphos	LC	27	Fenoxycarb	LC	82	Methoxyfenozide	LC	137	Tebufenocide	LC	192
Chlorobenzilate	GC	28	Fenpropathrin	LC	83	Metrafenone	LC	138	Tebufenpyrad	LC	193
Chlorpropham	GC	29	Fenpropimorph	LC	84	Mevinphos (phosdrin) (two isomers)	LC	139	Teflubenzuron	GC	194
Chlorpyrifos	GC	30	Fenpyroximate	LC	85	Monocrotophos	LC	140	Tefluthrin	GC	195
Chlorpyrifos methyl	GC	31	Fenthion	LC	86	Myclobutanil	LC	141	Telodrin (isobenzan)	GC	196
Chlorthal dimethyl	GC	32	Fenthion oxon	LC	87	N,N-Dimethyl-N'-p-tolylsulphamide (DMST,metabolite of tolylfluanid)	LC	142	Terbufos	GC	197
Clofentezine	LC	33	Fenthion oxon sulfone	LC	88	N,N-dimethylformamidine (DMF, metabolite of amitraz)	LC	143	Terbuthylazine	LC	198
Clothianidin	LC	34	Fenthion oxon sulfoxide	LC	89	Nuarimol	LC	144	Tetrachlorvinphos	LC	199
Coumachlor	LC	35	Fenthion sulfone	LC	90	Ofurace	LC	145	Tetraconazole	LC	200
Coumaphos	LC	36	Fenthion sulfoxide	LC	91	Omethoate	LC	146	Tetradifon	GC	201
Cyazofamid	LC	37	Fenvalerate	GC	92	Oxadixyl	LC	147	Tetramethrin	GC	202
Cyflufenamid	LC	38	Fipronil	LC	93	Oxamyl	LC	148	Thiacloprid	LC	203
Cyfluthrin (sum of four isomers)	GC	39	Fipronil sulfide	GC	94	Oxamyl oxime	LC	149	Thiamethoxam	LC	204
Cyhalothrin (lambda isomer)	LC	40	Fluazinam	LC	95	Oxyfluorfen	GC	150	Thiodicarb	LC	205
Cymoxanil	LC	41	Flubendiamide	LC	96	Paclobutrazol	LC	151	Tolclofos methyl	GC	206
Cypermethrin (sum of four isomers)	GC	42	Flucythrinate (two isomers)	GC	97	Paraoxon methyl	GC	152	Tolylfluanid	GC	207
Cyproconazole (two isomers)	LC	43	Fludioxonil	LC	98	Parathion ethyl	GC	153	Triadimefon	LC	208
Cyprodinil	GC	44	Flufenoxuron	LC	99	Parathion methyl	GC	154	Triadimenol	LC	209
Deltamethrin	GC	45	Fluopyram	LC	100	Penconazole	LC	155	Triazophos (hostathion)	LC	210
Demeton-S-methyl	LC	46	Fluquinconazole	LC	101	Pencycuron	LC	156	Trichlorfon	LC	211
Demeton-S-methyl-sulfone (Dioxydemeton)	LC	47	Flusilazole	LC	102	Pendimethalin	LC	157	Trifloxystrobin	LC	212
Diazinon	GC	48	Flutolanil	LC	103	Permethrin (two isomers)	GC	158	Triflumizole	LC	213
Dichlofluanid	GC	49	Flutriafol	LC	104	Phosalone	LC	159	Triflumuron	LC	214
Dichloran	GC	50	Fluvalinate tau	LC	105	Phosmet	LC	160	Trifluralin	GC	215
Diethathyl ethyl	LC	51	Fonofos	GC	106	Phosmet oxon	LC	161	Triticonazole	LC	216
Diethofencarb	LC	52	Fosthiazate	LC	107	Phthalimide (metabolite folpet)	GC	162	Vinclozolin	GC	217
Difenoconazole	LC	53	Hexaconazole	LC	108	Pirimicarb	LC	163	Zoxamide	LC	218
Diflubenzuron	LC	54	Hexaflumuron	LC	109	Pirimiphos ethyl	LC	164			
Diflufenican	LC	55	Hexythiazox	LC	110	Pirimiphos methyl	LC	165			

a Gas chromatography (GC) or liquid chromatography (LC), both coupled with tandem triple quadrupole mass spectrometry.

**Fig. 1:**
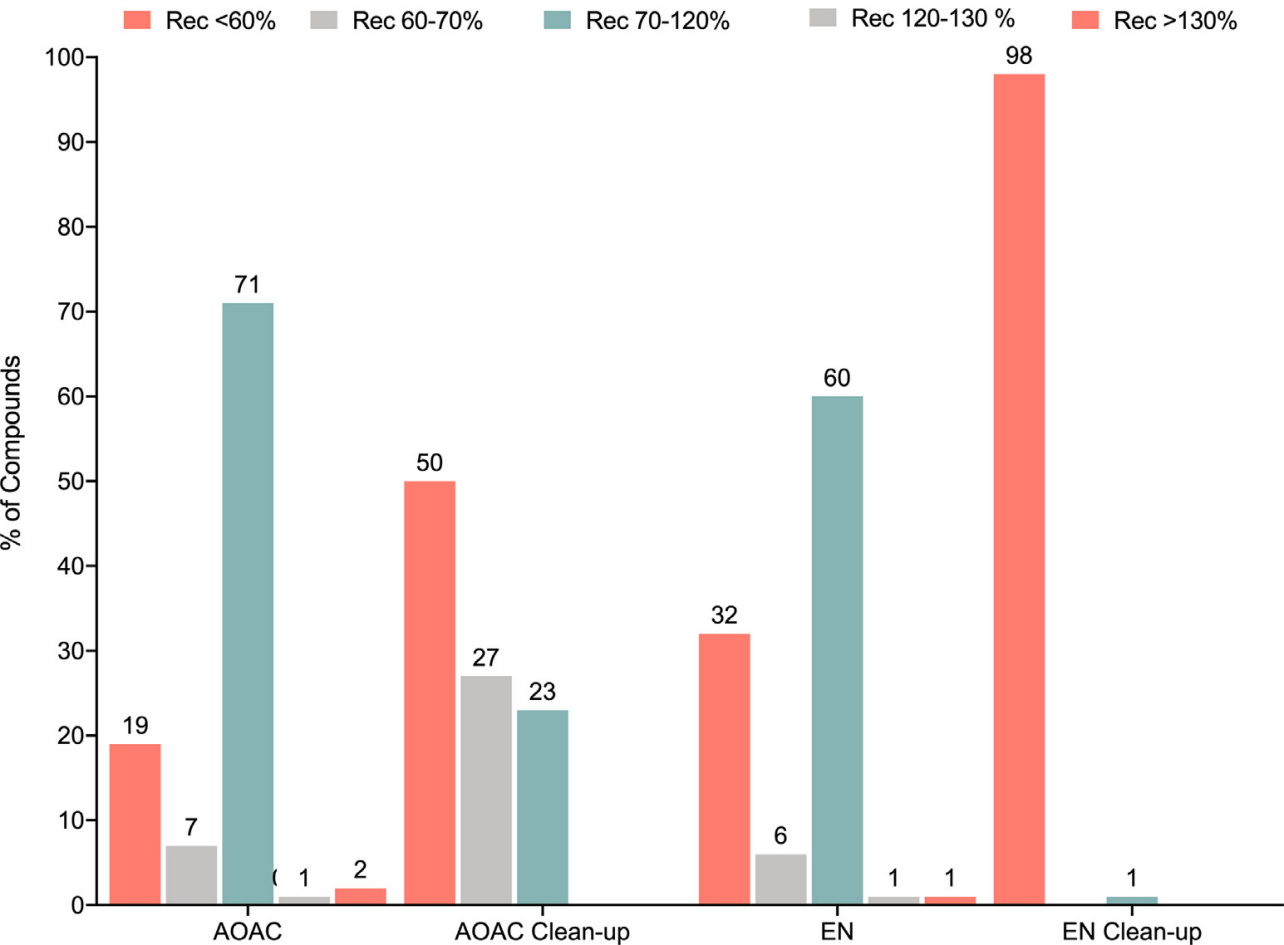
Performance of the four extraction methods tested with respect to recovery percentage. The figure shows for each extraction method the percentage of the 218 compounds that were recovered at <60%, between 60% and 70%, between 70% and 120% (optimal according to the SANTE guide), between 120% and 130%, and >130% the theoretical level of fortification.

Fig. 1 represents the percentage of compounds against the recovery (%) for AOAC and EN QuEChERS methods, with and without a clean-up step. For recoveries between 70% and 120%, analytes are considered to be successfully extracted under the SANTE 12,682/2019 and the SANCO 825/00 Rev.1 guidance document on residue analytical methods guidelines [2,3], which were followed for the optimization and validation processes. Recoveries in the ranges of 60–70% and 120–130% were considered as well, since further improvement can be achieved during the whole optimization procedures. According the mentioned guides, poor recoveries were considered below 60% and over 130%.

**Fig. 2:**
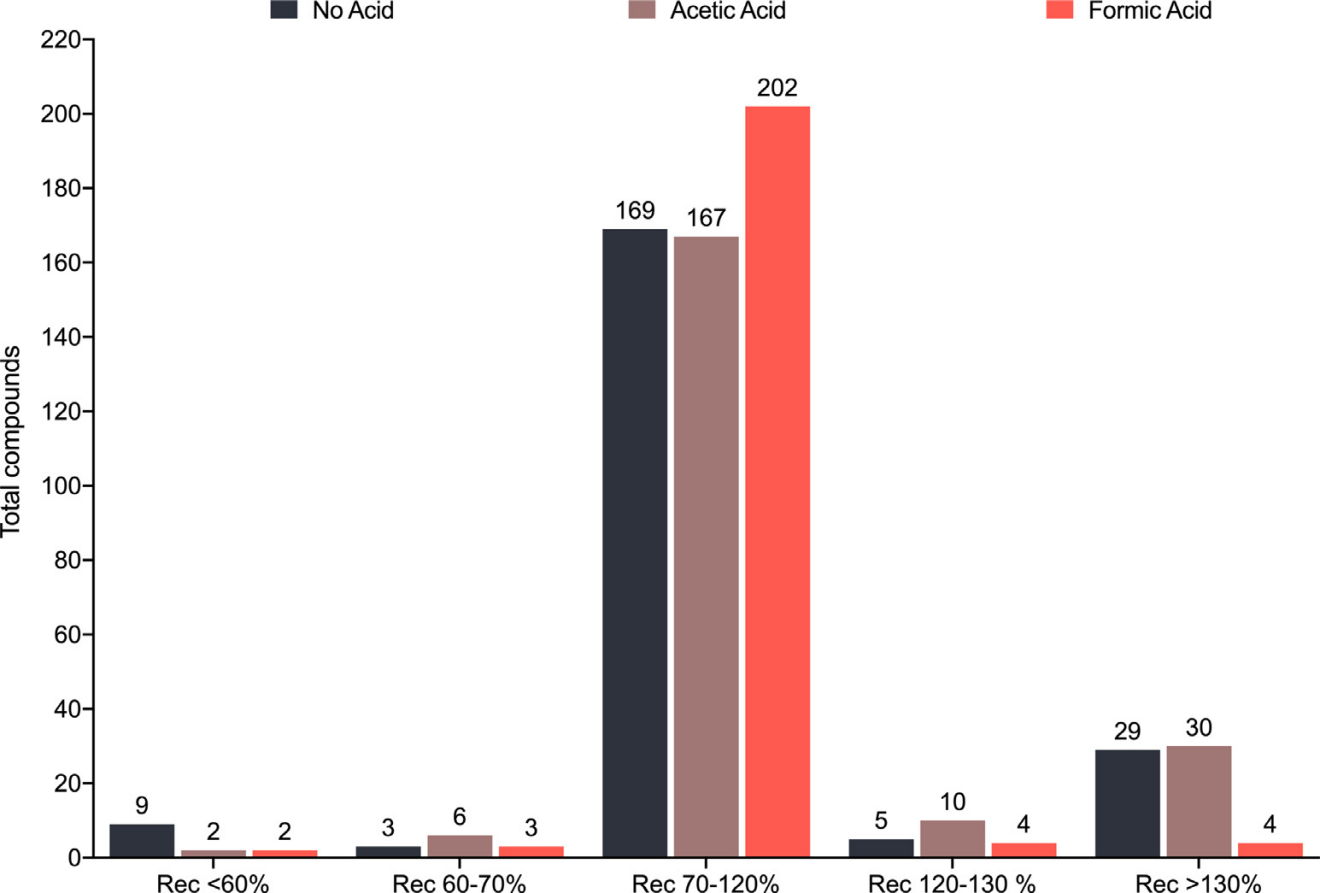
Acid addition to solvent extraction. The figure shows the number of compounds that, from left to right, had a recovery below 60%, in the range of 60% to 70%, between 70% and 120%, from 120% to 130%, and superior to 130% when the extraction solvent was acetonitrile (orange-coloured bars), acetonitrile-1% acetic acid (brown-coloured bars) and acetonitrile-1% formic acid (dark blue-coloured bars).(For interpretation of the references to color in this figure legend, the reader is referred to the web version of this article.)

In Fig. 2 we present graphically the results of the comparative study of the recovery percentages obtained for the 218 analytes when they are extracted in the presence of acid (either 1% formic acid or 1% acetic acid) or in the absence of it.

**Fig. 3:**
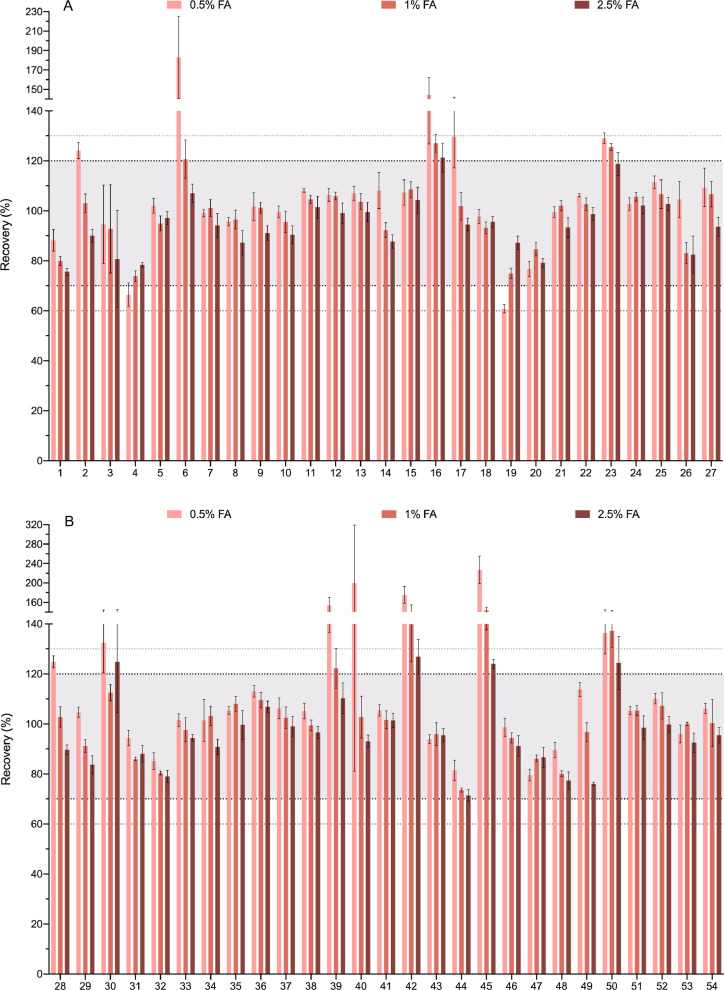

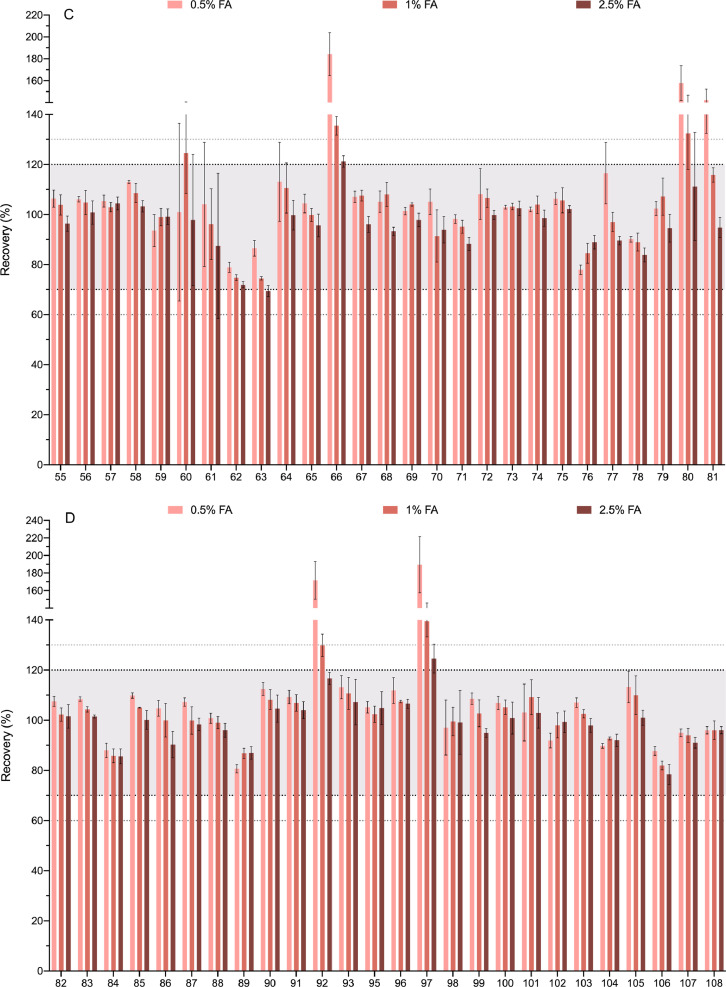

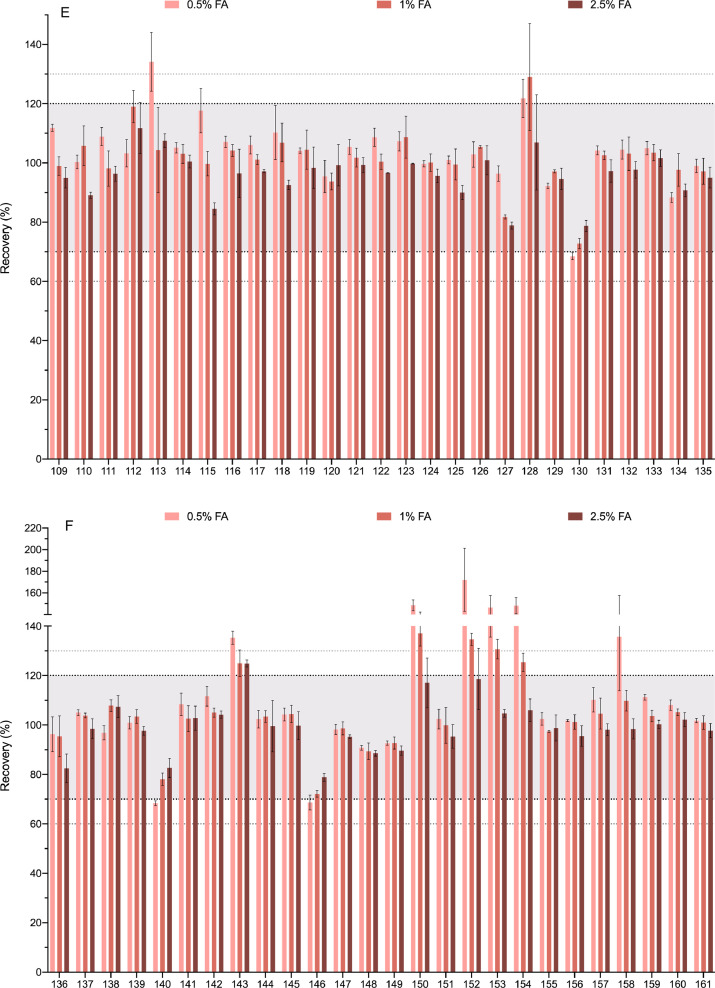

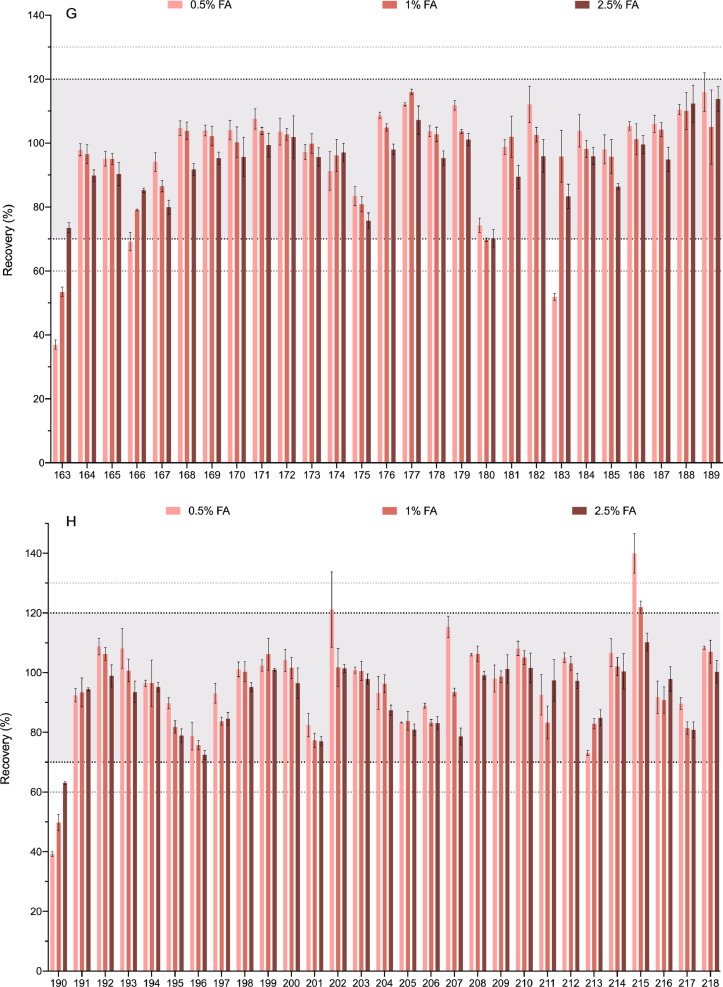
Optimization of the amount of formic acid. Three concentrations of F.A. (0.5%, 1%, 2.5%) are shown for each compound. The compounds are numbered according to the relation in Table 1. Bold-dotted lines shows the recovery limits recommended by the SANTE guide as acceptable (70% and 120%). Since the same guide also permits an expanded 60–130% when the results are highly reproducible these limits are also marked with dotted lines. For clarity the graph has been divided in 8 panels.

Fig. 3 shows the recovery of each compound when 0.5%, 1% and 2.5% of formic acid in the extraction solvent was tested. As stated above, analytes with recoveries between 70% and 120% (relative standard deviation (RSD)<=20, *n* = 3) were considered successfully extracted and that area is marked in the graphic. Ranges of 60–70% and 120–130% were also marked.

**Fig. 4:**
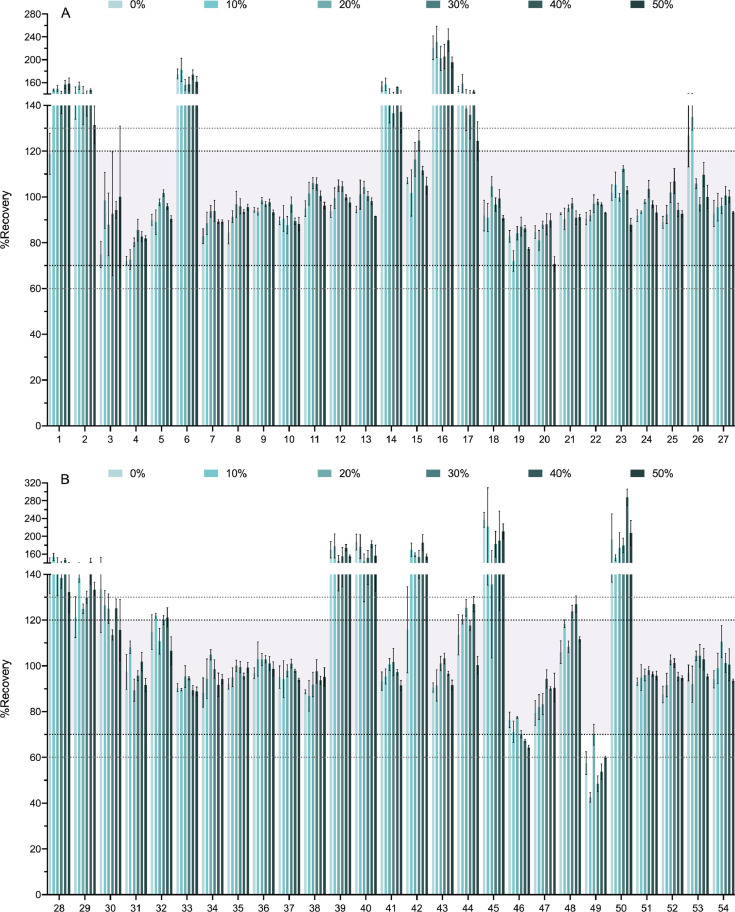

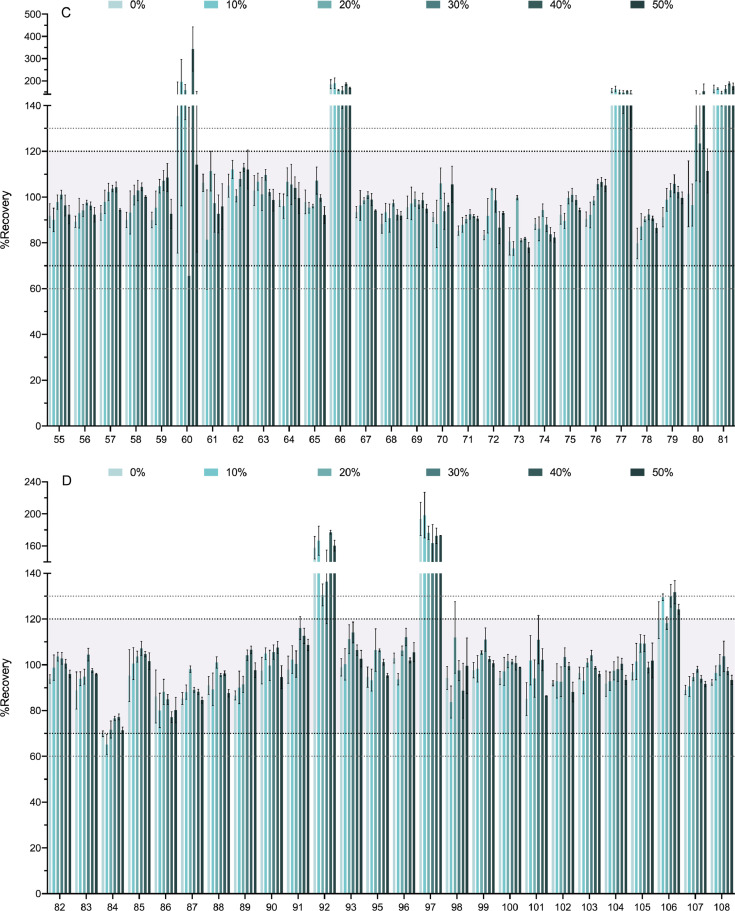

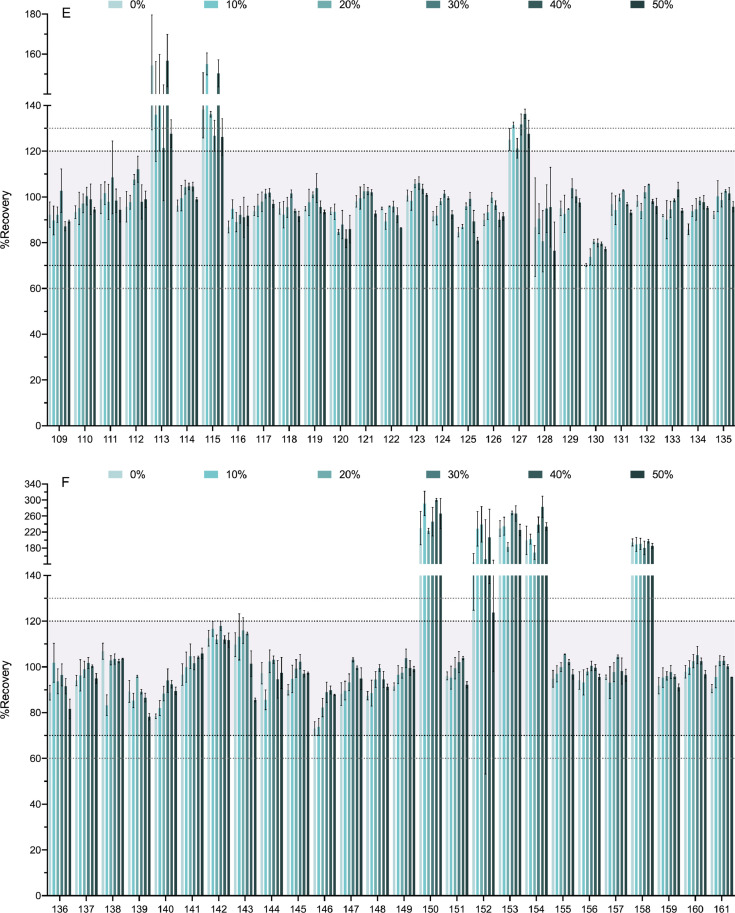

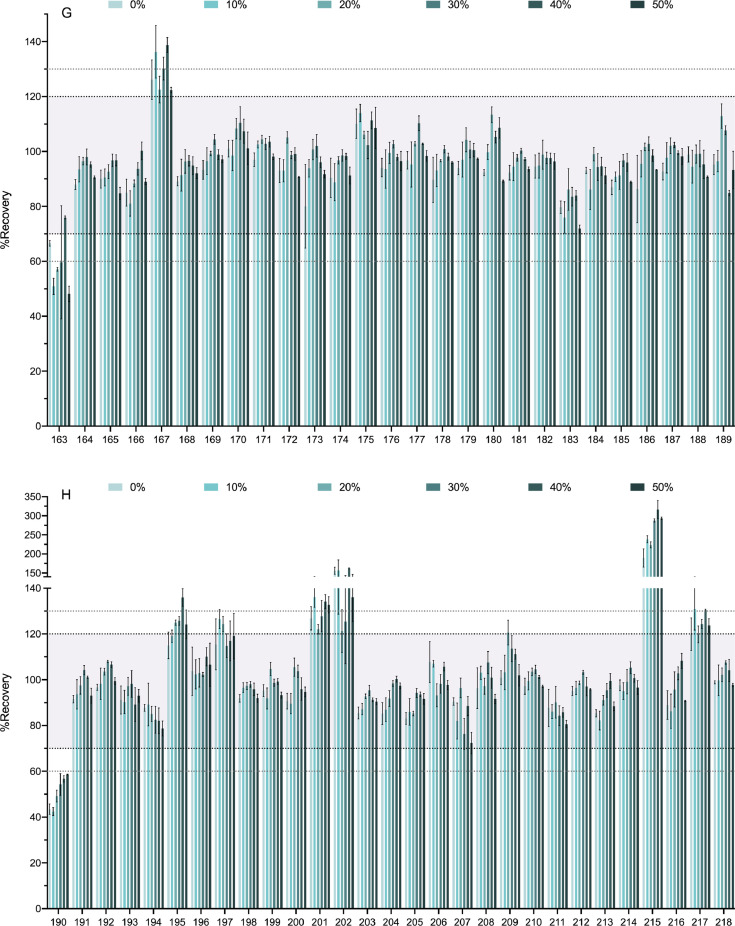
Soil sample moisture effect on the recovery percentages. In Fig. 4 we show the effect of soil moistening on the recovery percentages of the 218 analytes. Various percentages of soil moisture were tested (0%, 10%, 20%, 30%, 40%, and 50% water). The compounds are numbered according to the relation in Table 1. Bold-dotted lines shows the recovery limits recommended by the SANTE guide as acceptable (70% and 120%). Since the same guide also permits an expanded 60–130% when the results are highly reproducible these limits are also marked with dotted lines. For clarity the graph has been divided in 8 panels.

Fig. 4 shows the effect of the percentage of water added to the soil sample in the extraction recovery. Recoveries obtained for dry soil samples (0%) were compared to those obtained for 10%, 20%, 30%, 40% and 50% of moisture. The range from 70 to 120% of recovery is highlighted in the chart along with those of 60–70% and 120–130%.

**Fig. 5:**
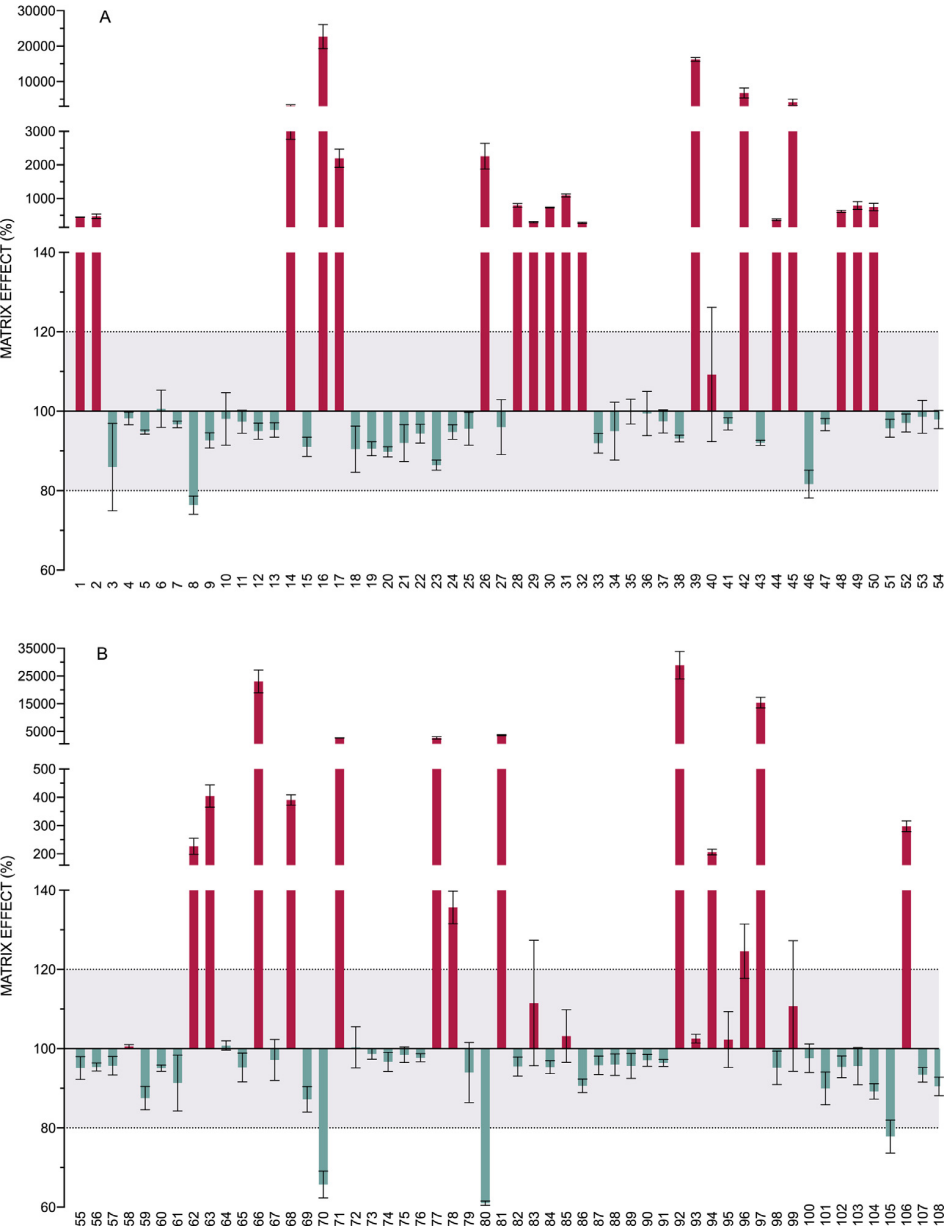

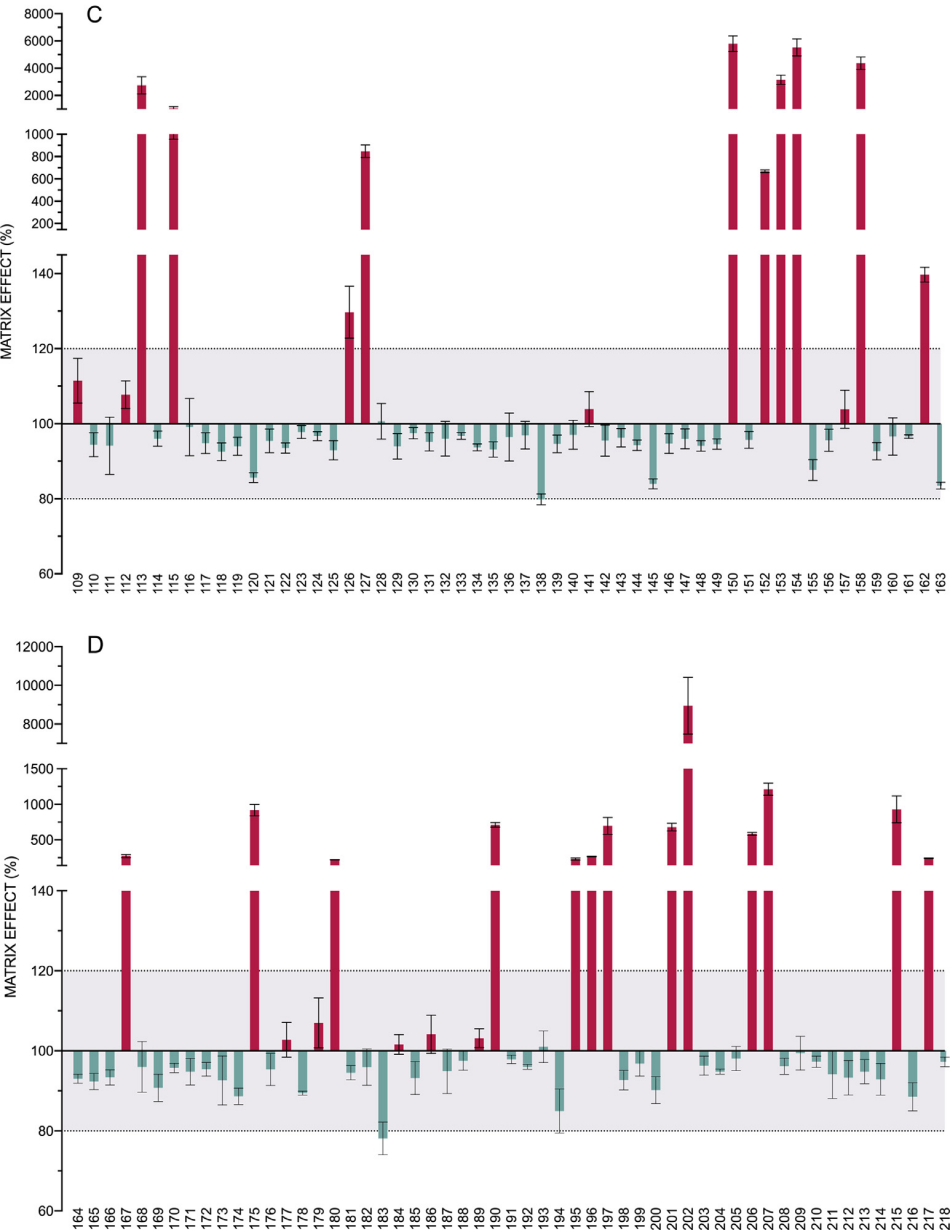
Matrix effect. Bars represent the mean and SD of the recoveries of the 218 analytes (spiked soil extract quantified against a calibration curve prepared in solvent). The compounds are numbered according to the relation in Table 1. Dotted lines represent the tolerance range in which it is considered that no significant matrix effect exists. For clarity the graph has been divided in 4 panels.

Fig. 5 is the representation of the matrix effect shown by each of the 218 analytes. It shows mean and SD values of ME in percentage for each pesticide and metabolite, which were calculated as follows: ME (%) =(*S_m_-S_b_*/*S_s_*) x 100, where *S_m_* is the signal obtained for each analyte in the soil extract, *S_b_* is the response of the non-spiked soil extract and *S_s_* is the signal of the standard in the solvent. The effect of the matrix components in the signal was rated as enhancement or suppression whether values of ME were above or below 100%, respectively. No significant matrix effect was considered if values were between 80% and 120. This range is marked in the graphic with a dotted line and the area that it covers had been shaded in grey.

The entire dataset of all these experiments are presented in the files named Fig. 1–5 raw data included as Supplementary Material of this article.

Finally, in Table 2 summarizes the rest of the method validation values, including LOD, LOQ, linearity, recoveries and reproducibility.

**Table 2:** Method validation results: LOD. LOD. linearity. recoveries and RSD.

						0.5 ng g^−1^		1 ng g^−1^		2.5 ng g^−1^		5 ng g^−1^		10 ng g^−1^		20 ng g^−1^		50 ng g^−1^	
No.	Compound	Technique	LODng g^−1^	LOQng g^−1^	Linearity	%REC	%RSD	%REC	%RSD	%REC	%RSD	%REC	%RSD	%REC	%RSD	%REC	%RSD	%REC	%RSD
1	4.4′-Dichlorobenzophenone (metabolite of dicofol)	GC	0.390	0.5	0.9975	115.0	8.2	118.1	4.0	112.4	7.7	106.2	3.3	99.4	5.0	95.3	7.7	100.6	2.8
2	4.4′-Dicofol	GC	3.125	20.0	0.9945	N/A	N/A	N/A	N/A	N/A	N/A	N/A	N/A	N/A	N/A	110.7	5.2	97.7	7.2
3	Abamectine	LC	3.125	20.0	0.9983	N/A	N/A	N/A	N/A	N/A	N/A	N/A	N/A	N/A	N/A	85.2	15.0	72.7	10.8
4	Acephate	LC	0.390	0.5	0.9990	90.5	6.2	83.2	2.8	69.3	4.1	65.3	3.6	66.3	2.4	62.2	8.2	65.7	1.1
5	Acetamiprid	LC	0.780	1.0	0.9940	N/A	N/A	106.4	10.7	90.6	5.7	83.1	5.7	93.0	4.9	90.7	5.1	89.0	3.8
6	Acrinathrin	LC	3.125	5.0	0.9963	N/A	N/A	N/A	N/A	N/A	N/A	93.0	13.6	90.9	8.8	85.9	26.6	91.2	9.2
7	Aldicarb	LC	0.024	0.5	0.9931	79.4	8.2	85.3	9.4	85.4	6.0	95.3	3.5	97.8	2.3	95.6	3.9	98.3	1.1
8	Aldicarb sulfone	LC	0.390	1.0	0.9993	N/A	N/A	111.8	6.5	80.4	6.4	83.9	3.6	84.8	5.5	81.0	4.7	79.3	2.4
9	Atrazine	LC	0.048	1.0	0.9958	N/A	N/A	91.5	9.5	66.4	8.9	98.3	5.6	101.9	1.5	95.1	0.9	93.5	1.5
10	Azinphos methyl	LC	0.097	0.5	0.9967	106.7	16.5	120.6	14.6	85.5	13.4	94.5	5.4	94.4	7.2	90.4	4.5	93.9	4.0
11	Azoxystrobin	LC	0.048	0.5	0.9952	104.3	7.8	109.1	8.0	90.3	1.6	99.1	4.2	103.6	4.0	95.2	5.3	94.7	1.7
12	Benalaxyl	LC	0.097	0.5	0.9953	109.0	8.2	106.9	3.0	91.1	3.7	101.4	4.2	101.9	2.0	96.2	3.9	94.0	1.9
13	Bendiocarb	LC	0.097	1.0	0.9965	N/A	N/A	113.5	2.2	91.7	3.6	101.0	6.5	103.5	3.9	97.5	3.6	96.8	2.1
14	Bifenthrin	GC	0.195	20.0	0.9973	N/A	N/A	N/A	N/A	N/A	N/A	N/A	N/A	N/A	N/A	122.7	7.1	118.8	2.5
15	Bitertanol	LC	0.780	2.5	0.9971	N/A	N/A	N/A	N/A	95.9	10.5	98.2	12.4	89.3	7.7	83.3	8.4	81.1	1.2
16	Boscalid (formely nicobifen)	GC	1.560	5.0	0.9950	N/A	N/A	N/A	N/A	N/A	N/A	116.6	12.7	99.7	13.8	97.9	14.3	98.4	4.6
17	Bromopropylate	GC	0.195	20.0	0.9980	N/A	N/A	N/A	N/A	N/A	N/A	N/A	N/A	N/A	N/A	130.7	11.0	130.7	2.7
18	Bromuconazole (two isomers)	LC	0.780	2.5	0.9995	N/A	N/A	N/A	N/A	82.3	19.6	85.1	5.5	91.9	6.3	88.0	5.1	83.9	5.8
19	Bupirimate	LC	0.097	0.5	0.9995	99.9	10.8	89.6	10.1	80.0	7.9	89.2	10.7	88.6	4.5	79.3	2.3	73.9	1.1
20	Buprofezin	LC	0.048	0.5	0.9993	83.4	9.4	91.4	5.5	79.7	5.8	93.8	3.8	90.4	2.3	84.9	2.4	76.7	3.3
21	Cadusafos (ebufos)	LC	0.048	0.5	0.9991	114.5	5.2	102.9	9.5	95.1	2.7	100.3	4.1	103.4	4.3	94.3	3.4	91.5	0.7
22	Carbaryl	LC	0.048	0.5	0.9997	95.5	7.2	90.5	6.9	82.7	4.2	88.6	4.4	93.8	2.0	87.3	2.5	87.3	1.4
23	Carbofuran	LC	0.048	0.5	0.9939	106.7	11.9	120.3	2.8	101.3	2.1	129.9	2.8	128.9	3.8	114.3	1.4	108.4	1.6
24	Carbofuran-3-hydroxy	LC	0.048	0.5	0.9954	102.5	18.0	101.0	9.5	89.6	4.2	97.4	4.0	102.4	4.1	94.3	2.2	91.5	1.0
25	Chlorantraniliprole	LC	0.390	1.0	0.9996	N/A	N/A	106.8	12.9	83.8	6.2	91.8	5.2	92.4	3.7	85.9	3.9	87.4	2.3
26	Chlorfenapyr	GC	1.560	10.0	0.9969	N/A	N/A	N/A	N/A	N/A	N/A	N/A	N/A	123.1	8.3	115.3	5.9	109.3	5.1
27	Chlorfenvinphos	LC	0.024	1.0	0.9986	N/A	N/A	104.7	14.0	103.7	10.4	110.0	5.9	108.1	3.5	101.7	1.9	93.6	2.9
28	Chlorobenzilate	GC	0.024	10.0	0.9976	N/A	N/A	N/A	N/A	N/A	N/A	N/A	N/A	117.0	4.5	114.3	8.6	115.0	3.3
29	Chlorpropham	GC	0.390	0.5	0.9997	112.5	15.3	130.2	8.3	122.1	6.4	112.1	4.2	106.7	2.1	100.5	5.5	105.6	3.5
30	Chlorpyrifos	GC	0.780	5.0	0.9985	108.3	9.1	N/A	N/A	N/A	N/A	109.9	7.8	101.7	5.8	98.7	13.1	105.9	7.6
31	Chlorpyrifos methyl	GC	1.560	5.0	0.9999	N/A	N/A	N/A	N/A	N/A	N/A	112.1	4.5	100.1	4.7	96.9	4.5	101.8	5.1
32	Chlorthal dimethyl	GC	0.048	2.5	0.9999	N/A	N/A	N/A	N/A	N/A	N/A	110.3	4.6	102.9	4.4	100.0	5.2	100.5	3.5
33	Clofentezine	LC	0.195	2.5	0.9901	N/A	N/A	N/A	N/A	85.7	11.8	115.9	5.2	115.5	3.9	106.1	5.3	95.1	3.5
34	Clothianidin	LC	0.780	2.5	0.9984	N/A	N/A	N/A	N/A	91.4	17.6	78.3	8.8	84.7	10.6	84.9	6.2	84.9	2.2
35	Coumachlor	LC	0.390	0.5	0.9915	91.7	19.1	91.1	14.4	91.1	7.2	99.9	11.0	114.9	5.3	102.2	5.2	100.8	2.2
36	Coumaphos	LC	0.195	1.0	0.9985	N/A	N/A	105.4	9.1	90.8	10.0	105.7	9.0	103.8	3.5	94.0	5.4	91.5	2.9
37	Cyazofamid	LC	0.780	5.0	0.9956	N/A	N/A	N/A	N/A	60.5	4.1	89.9	1.8	98.4	2.3	91.6	4.1	91.9	2.8
38	Cyflufenamid	LC	0.390	2.5	0.9934	N/A	N/A	N/A	N/A	89.1	16.7	101.5	8.3	102.6	4.3	98.7	4.9	92.5	2.0
39	Cyfluthrin (sum of four isomers)	GC	1.560	10.0	0.9997	N/A	N/A	N/A	N/A	N/A	N/A	N/A	N/A	112.1	7.4	113.0	9.8	99.6	6.1
40	Cyhalothrin (lambda isomer)	LC	6.250	20.0	0.9943	N/A	N/A	N/A	N/A	N/A	N/A	N/A	N/A	N/A	N/A	88.3	18.9	87.6	8.6
41	Cymoxanil	LC	0.390	0.5	0.9984	117.1	14.4	106.2	10.7	89.6	9.6	90.6	8.2	93.0	3.6	87.3	2.8	90.0	2.7
42	Cypermethrin (sum of four isomers)	GC	1.560	5.0	0.9971	N/A	N/A	N/A	N/A	N/A	N/A	95.0	16.4	84.2	9.7	88.0	12.6	88.8	3.3
43	Cyproconazole (two isomers)	LC	0.390	0.5	0.9957	105.4	10.8	101.0	3.2	90.8	7.5	93.3	7.5	96.4	2.4	92.8	3.0	85.9	1.5
44	Cyprodinil	GC	0.195	2.5	0.9999	N/A	N/A	N/A	N/A	102.3	10.1	93.4	3.6	87.6	4.4	86.2	7.1	86.1	1.1
45	Deltamethrin	GC	3.125	5.0	0.9990	N/A	N/A	N/A	N/A	N/A	N/A	115.9	11.3	90.9	16.9	94.8	10.7	80.0	6.7
46	Demeton-S-methyl	LC	0.195	0.5	0.9957	85.7	8.1	77.3	6.8	75.1	3.9	73.7	5.1	79.8	4.6	78.2	3.0	84.4	4.3
47	Demeton-S-methyl-sulfone (Dioxydemeton)	LC	0.097	0.5	0.9964	77.7	14.4	83.6	4.9	68.5	4.8	75.7	6.5	83.3	2.1	81.0	4.3	83.4	1.4
48	Diazinon	GC	0.195	0.5	0.9994	120.2	8.4	130.3	7.3	117.2	6.2	104.1	2.1	95.0	7.0	93.7	6.8	98.0	2.9
49	Dichlofluanid	GC	0.780	2.5	0.9992	N/A	N/A	N/A	N/A	84.0	9.5	104.9	7.3	96.9	7.0	65.5	4.6	73.1	5.1
50	Dichloran	GC	0.780	2.5	0.9994	N/A	N/A	N/A	N/A	87.2	18.2	99.6	26.2	81.4	19.7	74.0	5.2	60.0	6.6
51	Diethathyl ethyl	LC	0.048	0.5	0.9980	106.1	8.1	102.7	6.7	90.4	12.1	101.5	4.0	101.4	2.3	93.6	2.4	90.8	1.2
52	Diethofencarb	LC	0.097	20.0	0.9951	N/A	N/A	N/A	N/A	N/A	N/A	N/A	N/A	N/A	N/A	83.3	3.7	96.4	1.4
53	Difenoconazole	LC	0.390	1.0	0.9996	N/A	N/A	101.2	13.0	79.0	7.4	90.9	6.4	92.9	3.1	86.5	1.6	85.0	2.3
54	Diflubenzuron	LC	0.390	1.0	0.9931	N/A	N/A	77.6	5.1	73.3	10.5	102.8	7.3	105.7	9.1	107.7	5.1	97.7	2.0
55	Diflufenican	LC	0.195	0.5	0.9973	120.7	10.2	101.3	16.2	89.9	5.1	103.2	6.1	110.4	5.8	98.9	2.6	94.4	2.6
56	Dimethenamide	LC	0.195	0.5	0.9953	105.9	5.3	103.5	2.8	92.8	7.2	102.1	4.7	103.3	4.0	95.6	2.7	92.5	1.4
57	Dimethoate	LC	0.097	0.5	0.9980	86.8	12.3	90.8	5.1	88.4	7.1	95.4	8.3	97.7	3.6	92.0	4.0	89.7	1.2
58	Dimethomorph (two isomers)	LC	0.195	1.0	0.9992	N/A	N/A	91.4	3.9	89.1	12.4	90.4	7.6	96.0	5.3	87.5	7.1	90.9	2.3
59	Diniconazole-M	LC	0.780	1.0	0.9927	N/A	N/A	101.0	8.0	89.9	7.6	84.6	5.7	99.7	3.3	100.5	4.0	98.2	3.3
60	Dinocap	LC	6.250	20.0	0.9962	N/A	N/A	N/A	N/A	N/A	N/A	N/A	N/A	N/A	N/A	128.7	12.9	96.1	9.4
61	Diphenylamine	LC	3.125	20.0	0.9968	N/A	N/A	N/A	N/A	N/A	N/A	N/A	N/A	N/A	N/A	81.8	5.7	83.1	17.0
62	Endosulfan alfa	GC	0.390	0.5	0.9990	109.0	9.4	127.0	3.0	119.6	7.8	116.2	2.0	104.1	3.2	97.7	8.8	96.9	3.9
63	Endosulfan beta	GC	3.125	5.0	0.9970	N/A	N/A	N/A	N/A	N/A	N/A	117.8	0.8	105.8	1.8	101.5	8.2	102.2	4.0
64	EPN	LC	1.560	10.0	0.9988	N/A	N/A	N/A	N/A	N/A	N/A	N/A	N/A	99.1	7.3	93.5	3.6	94.1	2.6
65	Epoxiconazole	LC	0.195	1.0	0.9987	N/A	N/A	100.5	17.5	72.4	15.8	94.8	15.8	95.3	3.0	85.5	5.7	89.8	3.3
66	Esfenvalerate	GC	3.125	5.0	0.9986	N/A	N/A	N/A	N/A	N/A	N/A	102.3	2.7	90.8	6.4	85.1	9.0	82.7	5.0
67	Ethion (diethion)	LC	0.024	0.5	0.9989	104.4	7.8	104.4	4.3	90.7	6.4	97.3	4.1	101.3	3.9	93.0	2.2	91.9	2.0
68	Ethofumesate	GC	0.390	5.0	0.9996	N/A	N/A	N/A	N/A	N/A	N/A	114.1	6.0	99.8	8.1	96.4	10.7	100.2	2.0
69	Ethoprophos	LC	0.097	0.5	0.9961	85.4	13.8	80.6	13.8	87.3	13.9	101.1	4.0	100.5	3.4	98.0	2.4	95.1	1.4
70	Etofenprox	LC	0.390	1.0	0.9933	N/A	N/A	107.0	5.7	83.1	8.5	89.9	7.5	91.2	8.9	84.1	4.9	82.4	2.3
71	Etoxazole	LC	0.024	0.5	0.9918	111.2	3.3	107.3	7.2	85.5	7.2	88.3	2.6	89.4	4.1	81.3	7.5	82.4	2.3
72	Famoxadone	LC	1.560	2.5	0.9953	N/A	N/A	N/A	N/A	98.3	7.9	93.3	8.7	101.8	9.8	94.5	7.4	96.8	6.8
73	Fenamidone	LC	0.097	1.0	0.9973	68.9	12.6	81.9	12.2	76.9	7.6	81.1	6.5	79.1	5.7	76.4	3.8	82.7	1.8
74	Fenamiphos	LC	0.048	0.5	0.9996	94.3	5.4	89.1	7.8	84.7	2.2	90.1	4.4	92.5	2.5	82.1	3.7	87.8	2.3
75	Fenamiphos sulfone	LC	0.195	0.5	0.9991	95.5	9.6	99.0	3.2	83.4	6.0	92.7	3.7	94.9	2.2	89.6	3.5	88.1	1.0
76	Fenamiphos sulfoxide	LC	0.097	1.0	0.9996	N/A	N/A	102.4	6.8	85.0	5.1	86.6	3.4	88.6	4.1	86.0	1.1	81.1	0.7
77	Fenarimol	GC	0.048	10.0	0.9969	N/A	N/A	N/A	N/A	N/A	N/A	N/A	N/A	123.0	5.1	120.5	8.2	119.1	2.6
78	Fenazaquin	LC	0.097	0.5	0.9993	113.3	4.7	104.2	7.6	84.3	7.1	89.8	3.2	89.7	6.7	82.4	3.0	77.1	1.0
79	Fenbuconazole	LC	0.780	2.5	0.9988	N/A	N/A	N/A	N/A	84.8	7.8	89.6	13.1	96.7	5.4	88.9	7.3	90.4	3.6
80	Fenbutatin oxide	LC	0.780	2.5	0.9975	N/A	N/A	N/A	N/A	99.6	12.7	128.5	7.0	114.9	7.9	97.2	6.4	84.9	3.0
81	Fenitrothion	GC	3.125	10.0	0.9995	N/A	N/A	N/A	N/A	N/A	N/A	N/A	N/A	108.2	9.2	103.6	9.1	99.0	4.8
82	Fenoxycarb	LC	0.390	0.5	0.9978	101.2	11.7	101.0	5.7	91.8	7.2	97.0	5.4	99.9	7.1	94.1	2.8	92.0	2.4
83	Fenpropathrin	LC	0.780	1.0	0.9997	N/A	N/A	112.1	13.5	84.9	10.9	91.0	5.5	92.2	5.4	87.9	4.6	88.9	1.5
84	Fenpropimorph	LC	0.048	0.5	0.9952	96.5	3.0	87.9	7.5	71.6	4.1	79.2	2.2	81.5	4.0	74.4	4.6	75.9	1.6
85	Fenpyroximate	LC	0.048	0.5	0.9989	111.6	4.8	102.8	4.9	87.8	3.9	91.4	4.2	94.5	3.4	87.9	2.8	91.3	2.8
86	Fenthion	LC	0.390	2.5	0.9977	N/A	N/A	N/A	N/A	84.3	10.0	66.7	10.8	87.3	5.1	81.5	3.0	95.3	3.5
87	Fenthion oxon	LC	0.048	0.5	0.9957	90.0	5.0	86.5	6.1	80.9	3.5	90.3	3.8	93.4	4.0	84.9	3.3	90.2	1.5
88	Fenthion oxon sulfone	LC	0.390	0.5	0.9989	108.8	8.2	99.4	10.7	86.6	8.4	94.0	5.4	88.8	6.2	81.6	4.4	82.7	1.2
89	Fenthion oxon sulfoxide	LC	0.390	0.5	0.9989	95.7	7.3	101.4	9.9	77.7	11.0	92.4	3.8	89.2	4.3	84.9	2.1	78.1	2.4
90	Fenthion sulfone	LC	0.195	0.5	0.9996	117.6	10.8	106.3	6.3	91.1	6.2	86.5	9.8	91.3	3.2	87.4	3.9	90.6	2.1
91	Fenthion sulfoxide	LC	0.097	0.5	0.9989	101.0	9.8	104.3	7.6	93.1	3.1	102.6	4.7	105.2	3.8	99.6	2.4	91.2	1.7
92	Fenvalerate	GC	3.125	5.0	0.9984	N/A	N/A	N/A	N/A	N/A	N/A	103.1	5.2	89.1	5.8	81.9	10.8	80.6	6.0
93	Fipronil	LC	0.780	2.5	0.9976	N/A	N/A	N/A	N/A	83.5	18.2	93.2	18.5	95.2	9.4	98.1	4.7	100.6	8.1
94	Fipronil sulfide	GC	0.390	5.0	0.9998	N/A	N/A	N/A	N/A	N/A	N/A	111.1	3.8	106.9	5.0	105.3	7.7	104.1	2.7
95	Fluazinam	LC	0.390	2.5	0.9927	N/A	N/A	N/A	N/A	101.9	17.2	96.2	7.5	104.1	7.0	96.6	3.0	100.2	4.3
96	Flubendiamide	LC	1.560	2.5	0.9938	N/A	N/A	N/A	N/A	89.2	8.8	83.4	7.5	97.5	7.3	94.9	2.4	92.1	1.7
97	Flucythrinate (two isomers)	GC	0.780	5.0	0.9995	N/A	N/A	N/A	N/A	N/A	N/A	122.8	4.0	108.7	6.9	107.9	10.5	102.3	3.4
98	Fludioxonil	LC	1.560	5.0	0.9910	N/A	N/A	N/A	N/A	N/A	N/A	81.3	16.8	86.3	18.1	82.8	24.3	98.1	6.5
99	Flufenoxuron	LC	0.195	0.5	0.9931	103.4	12.9	105.5	8.0	91.1	6.0	89.8	3.2	92.7	3.8	81.8	4.2	80.4	2.6
100	Fluopyram	LC	0.195	0.5	0.9961	94.0	18.7	106.2	9.3	90.8	4.2	100.0	5.9	103.4	2.8	97.2	2.0	92.2	3.7
101	Fluquinconazole	LC	0.780	2.5	0.9903	N/A	N/A	N/A	N/A	100.9	16.0	97.0	8.1	91.5	11.1	91.2	5.3	94.7	5.1
102	Flusilazole	LC	0.195	1.0	0.9944	69.4	8.4	90.9	18.2	81.2	12.3	103.1	6.0	108.6	4.3	98.7	3.0	90.8	2.3
103	Flutolanil	LC	0.024	0.5	0.9934	98.9	14.5	95.9	10.1	94.7	9.2	108.8	4.4	110.6	3.1	99.8	2.4	94.1	1.2
104	Flutriafol	LC	0.780	1.0	0.9991	N/A	N/A	117.2	5.8	89.1	4.7	86.9	1.9	92.1	3.2	87.4	2.9	85.6	1.4
105	Fluvalinate tau	LC	1.560	2.5	0.9995	N/A	N/A	N/A	N/A	81.3	7.8	107.5	4.3	93.2	5.2	73.7	20.6	79.1	4.3
106	Fonofos	GC	0.390	0.5	0.9997	116.4	9.2	123.3	5.6	116.2	7.0	104.8	5.6	96.4	2.3	94.0	6.8	100.2	4.6
107	Fosthiazate	LC	0.024	0.5	0.9923	109.9	4.0	100.0	4.9	85.5	2.5	94.7	2.5	98.4	1.6	91.6	2.5	91.0	1.0
108	Hexaconazole	LC	0.390	2.5	0.9917	N/A	N/A	N/A	N/A	83.4	13.9	100.7	5.5	105.6	10.7	96.2	4.2	88.3	6.2
109	Hexaflumuron	LC	0.780	5.0	0.9922	N/A	N/A	N/A	N/A	N/A	N/A	99.0	7.0	104.3	7.2	93.1	5.9	82.9	4.3
110	Hexythiazox	LC	0.097	0.5	0.9993	115.1	4.5	105.1	6.0	83.2	4.5	93.5	8.5	95.1	3.7	88.2	3.6	89.4	2.1
111	Imidacloprid	LC	0.195	2.5	0.9988	N/A	N/A	N/A	N/A	75.2	14.8	86.0	4.2	81.5	6.5	79.7	3.5	79.9	3.8
112	Indoxacarb	LC	0.097	1.0	0.9957	N/A	N/A	101.5	5.9	92.5	7.8	95.0	6.5	105.0	4.2	89.9	5.2	100.3	4.9
113	Iprodione	GC	1.560	10.0	0.9972	N/A	N/A	N/A	N/A	N/A	N/A	N/A	N/A	106.6	12.4	120.8	4.7	111.5	7.5
114	Iprovalicarb	LC	0.195	0.5	0.9992	111.0	3.5	104.9	2.4	91.4	3.4	98.9	2.1	98.4	3.7	91.9	3.2	91.6	1.2
115	Isocarbophos	GC	1.560	5.0	0.9999	N/A	N/A	N/A	N/A	N/A	N/A	113.0	4.0	105.1	4.4	104.3	6.1	106.9	3.0
116	Isofenphos methyl	LC	0.390	0.5	0.9951	85.2	12.0	92.9	17.7	86.5	9.6	91.6	11.3	101.9	6.3	97.0	6.0	96.5	4.6
117	Isoprothiolane	LC	0.048	0.5	0.9988	76.0	13.0	88.5	7.3	86.4	4.4	100.7	4.6	100.3	3.7	94.6	1.9	89.6	2.4
118	Kresoxim methyl	LC	0.780	1.0	0.9973	N/A	N/A	90.5	13.9	106.4	16.9	91.3	10.6	96.6	9.2	93.3	4.6	88.4	5.3
119	Linuron	LC	0.780	1.0	0.9959	N/A	N/A	114.0	5.2	87.8	9.0	100.9	1.9	98.1	3.8	89.9	3.3	90.7	1.7
120	Lufenuron	LC	0.390	2.5	0.9926	N/A	N/A	N/A	N/A	97.7	6.5	108.7	3.6	113.9	4.6	103.6	3.1	91.4	3.2
121	Malaoxon	LC	0.048	0.5	0.9997	109.8	4.1	101.3	2.5	90.0	3.0	91.2	1.6	92.1	4.3	87.7	1.4	88.2	0.9
122	Malathion	LC	0.390	0.5	0.9960	101.7	9.0	108.7	7.9	91.7	4.9	103.9	2.4	105.0	1.1	98.0	3.9	94.4	0.8
123	Mandipropamid	LC	0.097	0.5	0.9989	109.4	4.4	104.0	5.0	91.5	6.1	97.3	1.8	98.9	1.5	93.1	3.2	90.6	2.4
124	Mefenoxam (metalaxyl-M)	LC	0.024	0.5	0.9995	98.2	6.0	98.8	3.5	86.2	4.8	90.3	3.5	91.5	3.0	85.9	2.6	84.3	1.0
125	Mepanipyrim	LC	0.780	1.0	0.9987	N/A	N/A	104.0	14.3	85.5	9.4	80.9	4.8	95.1	3.2	86.8	3.7	89.2	2.2
126	Metaflumizone	LC	0.024	0.5	0.9901	106.1	8.5	108.7	6.0	98.3	5.8	101.8	2.1	106.8	2.3	94.0	2.4	82.6	1.2
127	Metalaxyl	GC	0.195	0.5	0.9998	118.4	1.5	136.4	5.1	123.9	7.0	99.0	4.9	92.8	2.8	86.1	5.5	92.6	3.4
128	Metaldehyde	LC	6.250	20.0	0.9993	N/A	N/A	N/A	N/A	N/A	N/A	N/A	N/A	N/A	N/A	89.4	12.0	84.2	3.6
129	Metconazole	LC	0.097	0.5	0.9968	103.4	7.4	102.6	7.6	85.6	8.0	104.4	5.8	101.9	3.5	92.9	3.1	86.0	1.7
130	Methamidophos	LC	0.390	0.5	0.9994	101.8	7.5	85.0	5.8	66.9	3.0	66.7	2.9	65.9	2.5	60.0	5.4	63.7	2.4
131	Methidathion	LC	0.024	0.5	0.9919	100.8	1.6	104.1	6.9	89.4	6.6	102.8	3.3	104.8	3.4	97.8	2.8	97.3	2.1
132	Methiocarb	LC	0.195	0.5	0.9964	89.8	7.6	96.0	2.4	88.2	5.1	101.4	2.8	101.3	4.1	94.7	2.9	92.2	2.7
133	Methiocarb sulfone	LC	0.195	1.0	0.9995	N/A	N/A	113.1	9.3	94.8	4.5	91.3	6.0	96.1	6.6	85.5	6.2	88.8	2.7
134	Methiocarb sulfoxide	LC	0.195	0.5	0.9990	119.6	10.7	109.3	8.8	83.7	6.5	84.7	5.9	87.7	5.4	84.3	2.9	81.7	2.2
135	Methomyl	LC	0.195	1.0	0.9954	N/A	N/A	111.7	3.3	92.8	2.9	95.5	9.3	98.3	2.2	94.3	5.1	93.4	2.8
136	Methomyl oxime	LC	1.560	20.0	0.9961	N/A	N/A	N/A	N/A	N/A	N/A	N/A	N/A	N/A	N/A	72.3	4.6	72.7	8.8
137	Methoxyfenozide	LC	0.024	0.5	0.9949	113.8	1.9	106.1	4.4	93.8	4.3	100.2	3.5	102.0	5.0	96.1	3.2	94.4	2.3
138	Metrafenone	LC	0.780	2.5	0.9974	N/A	N/A	N/A	N/A	123.5	10.4	84.5	12.6	96.1	5.9	90.3	5.6	94.3	1.6
139	Mevinphos (phosdrin) (two isomers)	LC	0.097	0.5	0.9991	105.7	12.0	95.7	4.6	88.2	5.3	86.6	5.3	87.0	5.4	82.2	3.4	84.7	1.3
140	Monocrotophos	LC	0.195	0.5	0.9983	114.3	10.1	99.8	6.3	78.5	5.4	78.1	5.9	80.4	4.2	78.3	3.5	76.7	0.8
141	Myclobutanil	LC	0.390	2.5	0.9957	121.7	13.2	123.1	12.5	97.1	8.9	98.8	3.0	98.0	5.7	93.5	3.1	94.7	1.9
142	N.N-Dimethyl-N'-p-tolylsulphamide (DMST.metabolite of tolyfluanid)	LC	0.390	0.5	0.9951	110.1	6.5	107.4	4.7	105.8	2.9	100.0	5.9	105.8	3.6	106.2	3.6	102.5	2.0
143	N.N-dimethylformamidine (DMF. metabolite of amitraz)	LC	0.097	20.0	0.9916	N/A	N/A	N/A	N/A	N/A	N/A	N/A	N/A	N/A	N/A	114.5	3.5	115.1	0.6
144	Nuarimol	LC	0.780	2.5	0.9964	N/A	N/A	N/A	N/A	74.5	10.4	60.7	32.6	90.6	13.1	83.0	11.7	91.5	5.8
145	Ofurace	LC	0.024	0.5	0.9958	117.0	5.9	106.6	2.8	85.9	4.0	97.5	1.4	105.1	3.9	97.2	1.5	93.4	0.8
146	Omethoate	LC	0.097	0.5	0.9990	92.0	8.9	81.6	6.1	69.6	9.8	70.6	5.9	71.4	1.5	68.5	5.0	71.0	0.9
147	Oxadixyl	LC	0.390	0.5	0.9992	102.6	8.0	92.3	1.7	77.2	6.7	88.3	3.9	89.0	1.6	84.3	2.7	85.7	0.8
148	Oxamyl	LC	0.195	0.5	0.9998	92.8	5.5	90.5	7.9	80.0	4.3	82.1	2.9	83.8	4.4	78.9	4.0	78.8	1.0
149	Oxamyl oxime	LC	0.390	0.5	0.9980	97.6	16.0	92.8	9.2	76.9	3.3	85.9	2.4	88.9	4.2	84.4	2.7	85.5	1.1
150	Oxyfluorfen	GC	3.125	5.0	0.9957	N/A	N/A	N/A	N/A	N/A	N/A	123.6	9.7	107.6	7.5	98.1	8.8	73.8	24.5
151	Paclobutrazol	LC	0.780	1.0	0.9965	N/A	N/A	116.3	13.0	97.4	6.4	79.7	7.2	94.7	6.0	94.7	2.6	91.8	1.9
152	Paraoxon methyl	GC	1.560	5.0	0.9996	N/A	N/A	N/A	N/A	N/A	N/A	109.3	10.1	102.0	7.0	97.3	4.0	100.5	4.8
153	Parathion ethyl	GC	1.560	5.0	0.9981	N/A	N/A	N/A	N/A	N/A	N/A	98.7	9.1	94.0	9.6	89.3	11.0	85.8	3.8
154	Parathion methyl	GC	3.125	5.0	0.9976	N/A	N/A	N/A	N/A	N/A	N/A	111.9	1.6	100.0	7.5	92.7	5.8	91.0	3.4
155	Penconazole	LC	0.195	0.5	0.9967	118.6	9.9	100.0	18.1	88.6	4.6	99.6	6.0	101.0	4.1	93.9	2.1	91.7	2.0
156	Pencycuron	LC	0.390	0.5	0.9987	104.7	4.5	101.0	3.1	92.4	6.8	94.5	3.6	97.9	2.8	90.7	3.8	90.8	1.6
157	Pendimethalin	LC	0.780	2.5	0.9940	N/A	N/A	N/A	N/A	83.8	5.7	86.1	6.4	97.5	8.4	94.4	5.1	98.3	4.2
158	Permethrin (two isomers)	GC	1.560	5.0	0.9963	N/A	N/A	N/A	N/A	N/A	N/A	77.8	14.4	90.0	7.7	105.4	10.0	122.8	3.7
159	Phosalone	LC	0.097	0.5	0.9921	81.0	19.6	87.5	12.5	89.4	10.4	101.9	8.6	109.6	2.9	102.8	1.3	98.3	2.1
160	Phosmet	LC	0.195	0.5	0.9923	114.6	5.6	107.6	4.1	92.7	7.9	103.0	1.3	111.4	3.9	101.3	2.8	98.8	1.0
161	Phosmet oxon	LC	0.097	0.5	0.9964	97.3	1.7	89.8	4.2	81.4	4.6	92.6	2.3	94.0	3.7	87.6	5.0	88.7	0.9
162	Phthalimide (metabolite folpet)	GC	1.560	5.0	0.9997	N/A	N/A	N/A	N/A	N/A	N/A	117.6	10.4	104.0	6.4	90.0	5.5	95.9	1.9
163	Pirimicarb	LC	0.048	0.5	0.9927	101.9	3.6	85.5	1.1	64.2	4.1	73.9	2.4	74.8	4.1	71.2	3.1	67.0	1.4
164	Pirimiphos ethyl	LC	0.097	0.5	0.9992	102.5	12.0	102.1	5.6	86.6	4.2	98.5	2.3	95.9	4.0	89.5	2.7	86.8	1.8
165	Pirimiphos methyl	LC	0.097	0.5	0.9985	107.3	14.1	89.7	10.1	89.5	6.4	98.8	5.2	97.5	3.9	90.4	3.1	87.9	2.6
166	Prochloraz	LC	0.195	0.5	0.9923	120.3	19.5	108.9	4.9	73.8	10.4	90.9	8.2	100.2	5.5	90.5	4.8	85.7	3.6
167	Procymidone	GC	0.097	5.0	0.9992	N/A	N/A	N/A	N/A	N/A	N/A	110.4	3.1	105.4	3.1	105.2	12.9	105.0	4.2
168	Profenofos	LC	0.195	0.5	0.9979	98.4	9.2	100.2	6.4	83.7	3.1	93.7	6.4	100.9	3.5	92.8	3.8	92.7	1.6
169	Propargite	LC	0.024	0.5	0.9927	104.3	5.8	103.5	5.9	90.8	5.6	103.5	3.3	103.2	1.9	96.6	2.9	94.2	0.5
170	Propiconazole	LC	0.195	2.5	0.9979	N/A	N/A	N/A	N/A	109.2	14.6	89.9	10.4	100.5	6.4	92.3	4.5	88.9	2.8
171	Propoxur	LC	0.024	0.5	0.9958	100.1	2.3	99.5	4.3	90.6	5.0	101.5	2.7	104.4	4.3	98.0	2.1	97.1	1.2
172	Propyzamide (pronamide)	LC	0.390	1.0	0.9906	N/A	N/A	109.8	11.4	92.0	8.9	107.1	7.9	106.1	8.0	103.0	2.9	93.0	3.2
173	Proquinazid	LC	0.780	1.0	0.9991	N/A	N/A	116.5	2.4	89.3	5.1	95.4	9.3	92.8	3.0	83.3	3.6	85.0	1.7
174	Prothioconazole-desthio	LC	0.780	1.0	0.9949	N/A	N/A	117.5	6.0	89.4	4.1	88.2	7.1	94.6	8.7	89.0	4.2	87.3	3.2
175	Prothiophos	GC	0.780	5.0	0.9993	N/A	N/A	N/A	N/A	N/A	N/A	123.9	6.0	113.8	3.2	103.0	8.0	100.7	2.8
176	Pyraclostrobin	LC	0.097	0.5	0.9948	107.7	10.9	107.3	7.7	91.7	4.9	102.5	4.7	101.3	3.3	95.7	1.3	95.3	1.4
177	Pyrazophos	LC	0.390	1.0	0.9954	N/A	N/A	112.8	7.9	94.5	8.8	93.0	4.4	87.0	4.5	84.2	3.2	88.5	3.5
178	Pyridaben	LC	0.097	0.5	0.9989	102.5	3.5	103.1	2.2	91.2	5.3	98.2	4.6	97.0	3.7	92.2	2.4	90.4	2.4
179	Pyridaphenthion	LC	0.097	1.0	0.9947	N/A	N/A	104.0	19.6	89.3	6.3	101.6	3.4	101.9	3.8	93.4	3.2	96.0	3.1
180	Pyrimethanil	GC	0.195	0.5	0.9996	99.3	9.8	100.3	4.6	85.9	3.6	87.3	2.0	83.1	2.4	80.7	5.9	85.2	4.5
181	Pyriproxifen	LC	0.024	0.5	0.9907	113.4	5.2	108.4	3.8	91.3	5.0	103.3	2.4	103.8	2.5	98.1	3.4	95.5	0.6
182	Quinalphos	LC	0.390	1.0	0.9979	N/A	N/A	106.1	12.6	91.1	5.7	97.0	10.5	100.8	5.7	93.9	2.2	92.8	2.8
183	Quinoxyfen	LC	0.097	0.5	0.9981	90.1	16.5	86.8	14.3	81.1	9.1	97.3	7.4	95.5	6.0	92.1	2.9	81.1	5.4
184	Rotenone	LC	0.195	1.0	0.9931	N/A	N/A	106.5	19.9	90.0	10.9	83.5	6.0	87.8	8.4	89.3	3.1	96.6	2.0
185	Simazine	LC	0.195	0.5	0.9952	93.6	7.6	98.5	5.6	81.0	4.9	90.4	5.3	90.8	7.1	91.4	4.5	93.0	2.2
186	Spirodiclofen	LC	0.097	0.5	0.9961	112.6	6.9	108.4	1.6	89.4	6.6	102.1	3.6	101.8	2.7	96.8	3.3	94.7	1.0
187	Spiromesifen	LC	0.097	1.0	0.9988	N/A	N/A	111.3	6.7	88.8	7.9	100.9	7.0	111.2	9.1	101.6	5.1	97.3	7.1
188	Spirotetramat	LC	0.195	1.0	0.9988	N/A	N/A	102.1	7.3	86.7	12.5	84.5	4.9	92.0	7.4	83.6	4.7	87.4	2.6
189	Spirotetramat-enol	LC	1.560	5.0	0.9994	N/A	N/A	N/A	N/A	N/A	N/A	95.0	9.6	86.9	12.6	84.3	6.0	84.3	5.2
190	Spiroxamine (two isomers)	GC	1.560	2.5	0.9999	N/A	N/A	N/A	N/A	106.2	6.5	76.4	5.4	69.6	3.2	63.4	8.7	65.3	3.4
191	Tebuconazole	LC	0.780	2.5	0.9988	N/A	N/A	N/A	N/A	75.7	11.1	82.1	7.6	88.5	5.4	86.2	3.1	85.6	4.0
192	Tebufenocide	LC	0.024	0.5	0.9940	108.8	5.3	103.0	7.1	88.7	5.0	100.4	3.1	101.4	3.3	95.9	4.9	96.2	2.2
193	Tebufenpyrad	LC	0.390	0.5	0.9922	89.9	12.0	96.4	8.5	82.4	5.6	104.0	2.3	109.2	4.6	99.1	5.0	97.4	2.3
194	Teflubenzuron	GC	0.390	0.5	0.9993	89.6	11.6	99.6	4.1	99.0	6.3	102.2	5.3	99.1	2.5	93.6	2.8	97.1	3.5
195	Tefluthrin	GC	0.195	2.5	0.9996	N/A	N/A	N/A	N/A	117.4	5.0	108.1	2.8	101.8	3.7	99.1	1.3	101.1	1.2
196	Telodrin (isobenzan)	GC	0.780	2.5	0.9981	N/A	N/A	N/A	N/A	120.9	7.2	110.1	9.0	103.7	6.5	96.0	9.2	98.6	2.2
197	Terbufos	GC	0.195	2.5	0.9989	N/A	N/A	N/A	N/A	126.3	4.1	99.9	4.7	91.1	2.1	87.4	7.5	96.2	4.6
198	Terbuthylazine	LC	0.195	0.5	0.9907	101.7	3.3	98.9	1.7	91.7	4.6	106.9	3.2	108.6	3.7	98.3	3.4	92.3	1.0
199	Tetrachlorvinphos	LC	0.097	1.0	0.9957	N/A	N/A	104.5	12.7	83.7	13.2	102.9	5.7	105.6	4.0	96.7	3.6	91.9	3.5
200	Tetraconazole	LC	0.390	5.0	0.9977	N/A	N/A	N/A	N/A	N/A	N/A	90.1	11.3	93.9	5.6	93.2	4.7	88.1	5.7
201	Tetradifon	GC	0.780	2.5	0.9993	N/A	N/A	N/A	N/A	120.7	4.4	115.0	3.8	106.2	4.8	101.0	5.9	101.6	3.4
202	Tetramethrin	GC	1.560	5.0	0.9981	N/A	N/A	N/A	N/A	N/A	N/A	126.8	5.5	110.9	7.5	111.4	9.7	104.6	5.3
203	Thiacloprid	LC	0.390	0.5	0.9993	101.1	4.0	99.0	6.1	85.9	5.5	82.8	1.8	88.5	4.0	83.3	3.1	82.6	1.5
204	Thiamethoxam	LC	0.390	1.0	0.9973	N/A	N/A	87.0	7.9	83.9	4.6	85.7	11.5	91.2	5.6	91.9	8.4	90.5	3.5
205	Thiodicarb	LC	0.097	0.5	0.9999	101.9	2.8	94.5	5.8	79.8	2.4	82.9	3.1	83.6	2.6	77.6	2.8	75.8	1.9
206	Tolclofos methyl	GC	0.195	0.5	0.9992	113.6	12.6	131.1	3.8	123.2	7.0	109.7	4.8	100.0	2.6	94.3	8.0	100.3	4.8
207	Tolylfluanid	GC	1.560	2.5	0.9998	N/A	N/A	N/A	N/A	104.0	18.5	125.0	9.4	108.4	6.3	83.6	8.4	84.0	4.1
208	Triadimefon	LC	0.390	0.5	0.9971	102.3	10.7	109.4	6.8	92.8	11.1	99.3	7.4	97.6	2.6	97.1	1.8	95.5	1.9
209	Triadimenol	LC	0.048	2.5	0.9985	N/A	N/A	N/A	N/A	75.4	17.0	88.4	9.0	89.7	5.1	85.9	3.6	86.0	4.0
210	Triazophos (hostathion)	LC	0.097	0.5	0.9917	96.0	6.9	98.4	6.4	90.6	3.3	104.3	5.1	104.8	3.2	98.4	4.1	96.5	1.5
211	Trichlorfon	LC	0.780	1.0	0.9940	N/A	N/A	87.7	15.3	73.4	19.2	81.6	15.4	99.6	5.9	92.3	8.0	93.8	2.9
212	Trifloxystrobin	LC	0.024	0.5	0.9927	106.7	1.0	105.9	3.4	91.4	3.0	102.5	3.7	105.0	3.3	98.9	2.6	94.8	1.3
213	Triflumizole	LC	0.048	0.5	0.9986	101.0	4.5	93.5	8.9	82.0	5.0	91.1	4.4	90.9	5.1	84.9	2.0	79.2	2.2
214	Triflumuron	LC	0.195	0.5	0.9947	114.6	17.3	109.6	5.5	92.4	8.2	106.6	8.8	104.3	3.8	103.2	4.9	93.3	3.9
215	Trifluralin	GC	0.390	0.5	0.9991	100.2	9.3	106.4	7.8	104.8	12.4	113.2	8.8	93.4	6.4	87.1	9.3	84.2	9.8
216	Triticonazole	LC	0.048	2.5	0.9962	N/A	N/A	N/A	N/A	90.0	8.3	96.2	9.9	98.8	8.2	91.7	2.5	89.3	2.2
217	Vinclozolin	GC	0.195	0.5	0.9997	102.9	12.0	120.9	3.1	109.6	5.0	107.5	5.3	102.9	2.4	98.0	4.5	102.7	2.2
218	Zoxamide	LC	0.024	0.5	0.9979	97.1	15.2	95.8	17.6	94.7	11.9	92.6	7.1	98.4	8.2	93.6	4.1	89.6	3.5

## 2 Experimental Design, Materials and Methods

### 2.1 Chemicals, reagents and calibrators

The pesticide standards (purity > 97.1%) and procedural internal standards (P-IS) (Atrazine-d5, Carbendazim-d3, Chlorpyrifos-d10, Cyromazine-d4, Diazinon-d10, Linuron-d3 and Pirimicarb-d6) were obtained from CPA Chem (Stara Zagora, Bulgaria), Dr Ehrestorfer (Steinheim, Germany) and Sigma-Aldrich (Augsburg, Germany). All manipulation of the standards and procedures was done following the guidelines on good laboratory practice in residue analysis (www.fao.org/download/standards).

From individual stock standard solutions (1000 μg mL^−1^ in ACN) or commercial mixtures (10 μg mL^−1^ in ACN), an intermediate working mixed solution of 0.833 μg mL^−1^ was prepared. The P-IS mix working solution was prepared at 1 μg mL^−1^ in ACN. The working solutions were stored at −20 °C for a maximum period of 5 weeks, and employed to spike soil samples and to prepare calibration curves, either in matrix or solvent.

The QuEChERS salts were acquired in commercial premixes as it also was the Enhanced Matrix Removal-Lipid (EMR-lipid) (Agilent Technologies (Palo Alto, USA). All the solvents employed were of HPLC-MS/MS grade (Honeywell, Charlotte, USA). Ammonium acetate, formic acid and acetic acid were of the maximum purity available and acquired from Fisher Scientific (Loughborough, UK). Ultrapure water was prepared in the laboratory using a Gradient A10 Milli-Q System (Millipore, Bedfore, MA, USA).

### 2.2 Sample preparation

The extraction was based on the QuEChERS-based using 10 g of dried and sieved soil (with of without of increasing percentages of water), 10 mL of the extractant (ACN, ACN-1% acetic acid, ACN-0.5% formic acid, ACN-1% formic acid, or ACN-2.5% formic acid), and 7.5 g of the QuEChERS extraction salts mixture (either AOAC or EN formulas). The tubes were energetically shaken for 1 min, sonicated in an ultrasonic bath for 15 min, gently shaked for 25 min (rotary shaker), and centrifuged 10 min at 4200 rpm. Finally, supernatant was either filtered (0.2 µm) and directly analysed in GC–MS/MS or dissolved in milliQ water grade (1:1, v/v) and analysed in LC-MS/MS.

When it was necessary to spike the soil samples (either the 218 analytes plus P-IS, or P-IS alone) the samples were left to stand at least 1 h prior to extraction. All optimization experiments were made at a single concentration, 20 ng g^−1^ (in triplicate). Soil matrix for calibrators and matrix effect samples were extracted without any fortification.

### 2.3 QuEChERS salts selection

For these experiments we employed modifications of the QuEChERS (quick, easy, cheap, effective, rugged and safe) method, initially designed for the extraction of pesticides in fruits and vegetables [4]. The two main official variants of the original QuEChERS, the AOAC [5] and the EN variants [6], were compared. During this step, ACN was used as the extraction solvent. QuEChERS extraction salts used for each method consisted on 1.5 g of NaOAc and 6 g MgSO_4_ for AOAC version and 4 g MgSO_4_, 1 g NaCl, 1 g Sodium Citrate dihydrate and 0.5 g Sodium hydrogencitrate sesquihydrate for EN variant. Both methods were tested followed or not by an additional clean-up step using the Enhanced Matrix Removal sorbent (EMR, Agilent Technologies) [7]. Five mL of the supernatant were treated with 1 g of EMR, which had been previously activated with 5 mL of water. Then it was centrifugated and 3.5 g MgSO_4_ were added to 8 mL of supernatant to remove the remaining water. Finally, all extracts produced with AOAC, EN QuEChERS versions with and without clean-up were analysed by LC-MS/MS and GC–MS/MS [1].

### 2.4 QuEChERS optimization process

#### 2.4.1 Solvent acidification

Following the optimization of the salts, we tested the influence of the acidification of the acetonitrile in the extraction efficiency. First, it was necessary to decide the acidificant, and formic acid and acetic acid, both at 1% in ACN were assayed and compared with the non-acidified ACN extraction by the AOAC QuEChERS method without further purification. From this experiment we chose formic acid as the more appropriate, and then the optimal concentration of acid was selected from a set of experiments in which the concentrations of 0.5, 1 and 2.5% (v/v) were assayed. Finally, ACN-2.5% FA was chosen as the solvent.

#### 2.4.2 Water addition to the soil sample

The effect of the moisture of the sample was studied by adding different volumes of water to 10 g of soil sample prior to the extraction in order to achieve 10%, 20%, 30%, 40% and 50% (v/p). For this purpose, 1, 2, 3, 4, and 5 mL of ultrapure water were added to each sample 1 h before the extraction, once each sample was left to stand for another 1 h after the fortification with the pesticide and/or P-IS mixes.

#### 2.4.3 Matrix effect

For matrix effect experiments, 5 level calibration curves (0, 6.25, 12.5, 25 and 50 ng g^−1^) were prepared in solvent and matrix in triplicate. Soil matrix was extracted using the optimized, recommended procedure (AOAC salt combination, ACN-2.5% FA and air-dried soil samples). According to the technique they were going to be analysed by, curves in solvent were prepared in either ACN-2.5% FA or ACN-2.5% FA-H_2_O, 1:1 (v/v) and either in matrix or matrix- H_2_O, 1:1 (v/v) for GC–MS/MS and LC-MS/MS, respectively.

#### 2.4.4 Instrumental analyses

For the determination and quantification of the total amount of compounds, samples were analysed by liquid chromatography and gas chromatography, both coupled to triple quadrupole mass spectrometry. LC-MS/MS analyses were performed using a 1290 Infinity II LC System and a Triple Quad 6460 mass spectrometer (Agilent Technologies, Palo Alto, CA, USA). The Agilent Technologies, Poroshell 120 EC—C18 column (2.1 × 100 mm, 2.7 µm) equipped with a guard pre-column and pre-filter (2.1 × 5 mm, 1.8 µm) was used for the chromatographic separations. GC–MS/MS analysis were achieved with a GC System 7890B equipped with a 7693 Autosampler and Triple Quad 7010 mass spectrometer (Agilent Technologies). The chromatographic separation in GC was performed using two fused silica ultra-inert capillary columns Agilent HP-5MS (15 m x 0.25 mm i.d., 0.25 µm film thickness), connected by a purged union to allow the backflushing. A detailed description of the operation conditions, spectrometric parameters and the optimization procedure of both methods can be found in the main article [1].

#### 2.4.5 Method validation parameters

The validation of the developed method was performed following the recommendations of the European Union SANTE 12,682/2019 and the SANCO 825/00 Rev.1 guidance document on residue analytical methods (EC, 2010; EC, 2019b), which were followed in the absence of specific guidelines for the analysis of pesticide residues of pesticides in soil.

The linearity in the response was studied by injecting standards into the soil extract or in the soil extract diluted with water (1:1, v/v) in GC–MS/MS and LC-MS/MS, respectively, at nine concentration levels: 0.3, 0.5, 1.0, 2.5, 5, 10, 20, 50, and 100 ng mL-1. Accuracy (% recoveries) and precision (% relative standard deviation) were estimated by recovery experiments in spiked soil samples (in quintuplicate) at 7 concentration levels: 0.5, 1.0, 2.5, 5, 10, 20 and 50 ng g^−1^. Values were considered acceptable when recoveries were between 70% and 120% and RSDs ≤20%. The limit of quantification (LOQ) was set as the lowest concentration level that has acceptable accuracy and precision and the limit of detection (LOD) was selected as the lowest point of the calibration curve that meets all the agreements, had a signal-to-noise ratio (S/N) > 3 and an accuracy between 80% and 120%.

The confirmation of the identity of the analytes in the samples was performed with the acquisition of two MS/MS transitions, the quantification (Q) transition and the confirmation (q) transition, with a maximum ion ratio tolerance of ±30% and agreement of the retention time with a maximum deviation of ±0.1 min between the analyte in the sample and the reference standard. It should be noticed that for analytes with chiral isomers as cypermethrin, the sum of their isomers is provided as so is the residue definition. Nevertheless, when a single enantiomer is included in the residue definition, such as lambda-cyhalothrin, they were determined and quantified separately.

## Declaration of Competing Interest

The authors declare that they have no known competing financial interests or personal relationships which have, or could be perceived to have, influenced the work reported in this article.

## Appendix Supplementary materials

Image, application 1
